# Sparse/Low Rank Constrained Reconstruction for Dynamic PET Imaging

**DOI:** 10.1371/journal.pone.0142019

**Published:** 2015-11-05

**Authors:** Xingjian Yu, Shuhang Chen, Zhenghui Hu, Meng Liu, Yunmei Chen, Pengcheng Shi, Huafeng Liu

**Affiliations:** 1 State Key Laboratory of Modern Optical Instrumentation, College of Optical Science and Engineering, Zhejiang University, Hangzhou, Zhejiang, China; 2 Department of Mathematics, University of Florida, Gainesville, Florida, United States of America; 3 B. Thomas Golisano College of Computing and Information Sciences, Rochester Institute of Technology One Lomb Memorial Drive, Rochester, New York, United States of America; Institute of Psychology, Chinese Academy of Sciences, CHINA

## Abstract

In dynamic Positron Emission Tomography (PET), an estimate of the radio activity concentration is obtained from a series of frames of sinogram data taken at ranging in duration from 10 seconds to minutes under some criteria. So far, all the well-known reconstruction algorithms require known data statistical properties. It limits the speed of data acquisition, besides, it is unable to afford the separated information about the structure and the variation of shape and rate of metabolism which play a major role in improving the visualization of contrast for some requirement of the diagnosing in application. This paper presents a novel low rank-based activity map reconstruction scheme from emission sinograms of dynamic PET, termed as SLCR representing Sparse/Low Rank Constrained Reconstruction for Dynamic PET Imaging. In this method, the stationary background is formulated as a low rank component while variations between successive frames are abstracted to the sparse. The resulting nuclear norm and *l*_1_ norm related minimization problem can also be efficiently solved by many recently developed numerical methods. In this paper, the linearized alternating direction method is applied. The effectiveness of the proposed scheme is illustrated on three data sets.

## Introduction

Positron emission tomography (PET) holds one of the most important applications in nuclear medical imaging device as a biomedical research technique and clinical diagnostic procedure. A fundamental characteristic of the biological system is that its metabolism is inherently time-dependent. Thus, the ability of PET to observe physiological and biochemical processes in living subjects in a dynamic mode has potential to enhance our understanding of drug activity during preclinical drug development and diseases like kinds of tumors or cancers.

Dynamic PET imaging is usually performed with the collection of a series of frames of sinogram data taken at ranging in duration from 10 seconds to minutes. Earlier dynamic image reconstruction approaches largely fall into two groups. The first one attempts to reconstruct the activity maps in the same manner as static PET imaging. Iterative statistical methods have been a primary focus on many efforts, including notable examples of maximum likelihood-expectation maximization (ML-EM) [1–3], maximum a posteriori (MAP) [4–6], penalized weighted least-squares [7–10], and penalized-likelihood (SAGE) algorithms [11–13]. With the continuing progresses of PET imaging, much attention has also been paid on 3D PET reconstruction [14–17] and TOF-PET reconstruction [18, 19].

The second group attempts to improve the signal-to-noise ratio (SNR) by integrating the iterative statistical methods with prior temporal knowledge as reconstructing tokens and some recent works use the noise reduction technique. It includes the use of temporal voxel smoothing [20, 21] and temporal basis function [22]. On the other hand, there have been considerable efforts aimed at using time-varying filters. Some of the most interesting ideas include the use of wavelet filter [23, 24], the use of principal components transformation (also called as Karhunen CLove transform) [25, 26], and the use of a tensor product spline basis [27, 28].

Although of great progresses achieved, there are still something to be improved. In general, specific assumptions on the measurement distribution (Poisson or Shifted Poisson) is required in conventional methods. It results in a relative long acquisition time for each frames. Otherwise, when the acquisition time is not sufficient, the proportions of the scatter and random events in all events would increase, which generally leads to a poor visual image quality and a poor contrast of the target region in reconstruction images. In addition, the correlation between different frames was usually ignored in the prior techniques, and it leads to all of them are powerless to extract the useful motion or shape deformation information during reconstruction.

Inspired by the recently developed sparse and low rank representation, we develop a novel dynamic PET reconstruction model that aims at making full use of the information of the adjacent frames to achieve a high quality reconstruction without any specific assumptions on the measurement distribution. Following the robust principal component analysis [29, 30] paradigm, the background is formulated as a low rank component while variations between successive frames are abstracted to the sparse. Then, the linearized alternating direction method is applied to tackle the optimization problem with affine constraint of the PET imaging. To demonstrate the effectiveness and robustness of the proposed method, three experiments are designed and shown in this paper.

The rest of this paper is organized as follows. In section 2, problem formulations and solutions are presented. Section 3 provides experiments along with results compared with the previous methods.

## Materials and Methods

### Notations

In this section, a brief summary of the notation used in the following paper is given. Matrices are all capital, vectors are lowercase. For instances, *Y* is a matrix and *y* is a vector. The lowercase *i* represents the number of the frames of PET sinogram or images. Nuclear norm of matrix is denoted by ∥*X*∥_*_, defined as the sum of the input matrix singular values, it represents the rank of matrix. ∥*X*∥ denotes Frobenius (or Euclidean) norm and 〈*X*, *Y*〉 is the standard inner product, and ${∥X∥}_{F}^{2}=\langle X,X\rangle$. ∥*X*∥_1_ is *l*_1_-norm which represents the sum of the absolute value of all elements in a matrix, ∥*X*∥_0_ is *l*_0_-norm which represents the number of the non-zero elements in matrix.

### Basic Data Model for Dynamic PET Imaging

For non-dynamic PET imaging, the signal and reconstructed image are related by the following equation:

y=Gx+n (1)

Where *y* represents the sinogram (available data), *G* represents the detection probability, *x* is the reconstruction image and *n* is the noise. In dynamic imaging, a series of the temporal sinogram data are acquired. Denoted by *y*_*i*_ the sinogram from the *i* − *th* scan, and by *x*_*i*_ the image of the *i* − *th* frame. Stack each *x*_*i*_ and *y*_*i*_ as a column vector of matrix *X* and matrix *Y* respectively, that is:

$$X=\left[x_{1},x_{2},...,x_{i},...,x_{n}\right].$$ (2)

$$Y\;=\;\left[y_{1},y_{2},...,y_{i},...,y_{n}\right]=\left[Gx_{1}+n_{1},...,Gx_{n}+n_{n}\right]$$ (3)

with *G* the system matrix. Then the data model for dynamic PET imaging can be written as follows:

Y=GX+N, (4)

where *N* = [*n*_1_, *n*_2_, …, *n*_*i*_, …, *n*_*n*_].

### Sparse and Low rank Representation

Sparse and low rank representation, also widely known as robust principle component analysis techniques [29, 30] in image analysis or processing, is a novel concept in the medical imaging community. In recent years, some researchers have proposed some related methods in CT and MRI [31, 32]. However, it is the first time to be used in PET imaging. The separated background and dynamic information are useful for PET preclinical/clinical application like Image-guided radiation therapy (IGRT) [33]. But it is difficult to find the strict stationary or background component in PET imaging directly. Because PET images reveal the radio-activity distribution in body which means that all the regions in PET images are not constant. However, compared to the target region, the varying rate of the background component in PET images is relative low. Therefore, this component could be considered as an invariant component. However, this assumption would result in the sparsity of the dynamic PET data is not pronounced in some situations. To achieve high quality reconstruction images, a framelet domain transformation is used to constrain the sparsity during the reconstruction. This transformation not only ensures the mathematical constraint required by sparse and low rank representation, also enhances the tolerance of noise and data loss for reconstruction. It is quite helpful to obtain high contrast reconstruction images in the low count or under-sampling situation.

### Model description

Hence, we stack each frames in the sequence as a column to form a matrix *X*, and decompose it into two disjoint parts:

$$X=X_{1}+X_{2}$$ (5)

where *X*_1_ is the low-rank component of *X*, which models the stationary background (or reference state) over time, and *X*_2_ is the sparse component of *X*, which represents the variation in intensity from one frame to another. Now, rewriting the Eq (4) as:

$$Y=G\left(X_{1}+X_{2}\right)+N.$$ (6)

Based on the idea about decomposing the images matrix *X* into a low rank matrix and a sparse matrix, and the model Eq (6), the following matrix minimization problem is a natural choice for sparse and low rank representation model in Dynamic PET imaging:

$$\begin{array}{l} \left(X_{1},X_{2}\right)=\text{arg}\text{min}\left(rank\left(X_{1}\right)+\lambda {∥AX_{2}∥}_{0}\right)\\ +\frac{1}{2\tau }∥G\left(X_{1}+X_{2}\right)-Y{∥}_{F}^{2} \end{array}$$ (7)

Where *A* is a tight framelet transform operator, the sparsity of *X*_2_ is enhanced in the wavelet domain. *λ* > 0 and *τ* > 0 are parameters balancing the weights of the low rank matrix, sparse matrix, and reconstruction error in the decomposition. In the model Eq (7), ∥⋅∥_*F*_ is the Frobenius norm. ∥*AX*_2_∥_0_ is defined as the total number of non-zero elements in matrix *AX*_2_. By minimizing the first two terms, *X*_1_ and *X*_2_ are forced to be low rank and sparse respectively. The accuracy of the reconstruction could be improved by minimizing the last term in Eq (7). However, the model Eq (7) is non-convex and hard to solve. As suggested in the literatures [34] to make problem tractable, we consider the following relaxed problem of Eq (7):

$$\begin{array}{l} \left(X_{1},X_{2}\right)=\underset{X_{1},X_{2}}{\arg\min}{∥X_{1}∥}_{*}\\ +\lambda {∥AX_{2}∥}_{1}+\frac{1}{2\tau }{∥G\left(X_{1}+X_{2}\right)-Y∥}_{F}^{2}, \end{array}$$ (8)

Where ∥⋅∥_*_ is the nuclear norm. For a matrix *M* of dimension *m* × *n*, ${∥M∥}_{*}=:\sum_{i}^{\min(m,n)}\sigma_{i}$, and *σ*_*i*_ is the *i*-th largest singular value of *M*.

### Alogrithm

In this section, we give a description about the solution of the proposed model. To solve model Eq (8), Linearized Alternating Direction Method (LADM) is applied. Denoted by $L(X_{1},X_{2})$ the objective function in the proposed model Eq (8), i.e.,

$$\begin{array}{l} L\left(X_{1},X_{2}\right)=∥X_{1}{∥}_{*}\\ +\lambda ∥AX_{2}{∥}_{1}+\frac{1}{2\tau }{∥G\left(X_{1}+X_{2}\right)-Y∥}_{F}^{2} \end{array}$$ (9)

Then alternating direction scheme iterates as follow

$$\begin{array}{l} X_{1}^{k+1}=\arg\min_{X_{1}}L\left(X_{1},X_{2}^{k}\right)\\ =\arg\min_{X_{1}}∥X_{1}{∥}_{*}+\frac{1}{2\tau }{∥G\left(X_{1}+X_{2}^{k}\right)-Y∥}_{F}^{2} \end{array}$$ (10)

$$\begin{array}{l} X_{2}^{k+1}=\arg\min_{X_{2}}L\left(X_{1}^{k+1},X_{2}\right)\\ =\arg\min_{X_{2}}∥AX_{2}{∥}_{1}+\frac{1}{2\tau }{∥G\left(X_{1}^{k+1}+X_{2}\right)-Y∥}_{F}^{2} \end{array}$$ (11)

Where k is the number of the iterations. To solve the *X*_1_ subproblem we linearize the data fidelity term at $X_{1}^{k}$, that is

$$\begin{array}{l} ∥G\left(X_{1}+X_{2}^{k}\right){-Y∥}_{F}^{2}\approx ∥G\left(X_{1}^{k}+X_{2}^{k}\right){-Y∥}_{F}^{2}\\ +\langle g_{X_{1}}^{k},X_{1}-X_{1}^{k}\rangle +\frac{\beta_{1}}{2}{∥X_{1}-X_{1}^{k}∥}_{F}^{2} \end{array}$$ (12)

where $g_{X_{1}}^{k}=2G^{⊤}\left(G\left(X_{1}^{k}+X_{2}^{k}\right)-Y\right)$ is the gradient of the linearized data term at $X_{1}^{k}$ and *β*_1_ > 0 is a constant.

As for *X*_2_, by using the fact that *A* ^−1^ *A* = *I*, we linearize $∥G\left(X_{1}^{k+1}+X_{2}\right){-Y∥}_{F}^{2}$ at $AX_{2}^{k}$ as follows:

$$\begin{array}{l} ∥G\left(X_{1}^{k+1}+X_{2}\right){-Y∥}_{F}^{2}\approx ∥G\left(X_{1}^{k+1}+A^{-1}AX_{2}\right){-Y∥}^{2}\\ =\;G\left(X_{1}^{k+1}+A^{-1}AX_{2}^{k}\right)\;-\;Y+\;\langle g_{AX_{2}}^{k},AX_{2}-AX_{2}^{k}\rangle \\ +\;\frac{\beta_{2}}{2}\;{∥AX_{2}-AX_{2}^{k}∥}_{F}^{2}\; \end{array}$$ (13)

where $g_{AX_{2}^{k}}$ is the gradient of $∥G\left(X_{1}^{k+1}+X_{2}\right){-Y∥}_{F}^{2}$ at $AX_{2}^{k}$. After above linearization, the sub-problems of *X*_1_ and *X*_2_ can be transformed to the following standard forms with closed form solutions as follows respectively:

$$\begin{array}{c} US_{\epsilon }\left[S\right]V^{T}\;=\;\arg\min_{X}{\epsilon ∥X∥}_{*}+\frac{1}{2}{∥X-W∥}_{F}^{2}\\ and\;S_{\epsilon }\left[W\right]=\arg\min_{X}{\epsilon ∥X∥}_{1}+\frac{1}{2}{∥X-W∥}_{F}^{2}, \end{array}$$ (14)

Here, *W* is the remaind terms of optimal function, for example, when *X*_1_ is the desired matrix, terms including *X*_2_ will be the *W*, *USV* ^*T*^ is the Singular Value Decomposition(SVD) of *W* and for a matrix *W* $S_{\epsilon }\left[W\right]$ is also a matrix, and ${\left(S_{\epsilon }\left[W\right]\right)}_{i,j}=\max\{0,|w_{i,j}\left|-\epsilon \right\}sgn\left(w_{i,j}\right).$ We alternatively solve Eqs (12) and (13), but only do one iteration for each sub-problem. The program should be stopped when the relative stopping criterions (based on empirical estimations) are reached:

$$\begin{array}{l} \;\epsilon_{1}\;=\;\frac{∥X_{1}^{k+1}\;-\;X_{1}^{k}∥}{∥X_{1}^{k}∥}\;<\;10^{-4},\\ \;\epsilon_{2}\;=\;\frac{∥X_{2}^{k+1}\;-\;X_{2}^{k}∥}{∥X_{2}^{k}∥}\;<\;10^{-4}, \end{array}$$ (15)

The convergence of the scheme can be proved similarly as that in [35] if *β*_*i*_ ≥ ∥*G*∥^2^, (*i* = 1, 2).

### Parameters and convergence

Since constrain parameters have a great influence on the final results, they should be chosen carefully. In this work, *λ* is used to balance the low rank and sparse decomposition, its value will influence the proportion of the stationary and dynamic components in decomposition. Candes [29] has recommended that the most appropriate value of *λ* is expressed as $\lambda =\frac{1}{{\left(\max(n,m)\right)}^{1/2}}$, where *n*(*m*) is the number of rows (columns) of sinogram. The constants *β*_1_, *β*_2_ are viewed as the stepsizes for iterations, and affect the speed of convergence. In this work, the boundary of *β* is set from 0.1 to 10. The maximum number of iteration is set 1000 and iteration will be stopped if the stopping criterions Eq (15) are met.

## Results

Three experiments were designed to evaluate the effectiveness of the SLCR in this work. Dynamic PET data corresponding to Zubal-thorax, brain and cardiac were used in these experiments respectively. Monte Carlo simulation (using a toolbox GATE) was used to create the experimental data sets. All experiments are well designed and focus on distinguishing target region boundary (Zubal-thorax), accurate and high contrast and clear boundary image reconstruction for low count data (brain) and extracting dynamic and structural information respectively when the organ has a large deformation (cardiac).

Maximum likelihood expectation maximization (ML-EM, code is based on image reconstruction toolbox by Fessler) was used as the comparison in this work. The maximum iterative number of ML-EM is set to 100 in all three experiments. All codes in the three experiments are implemented in Matlab R2011a (MathWorks Corporation, USA) and run in a desktop computer with i3 Intel Core CPU and 4 GB memory.

In order to analyze the reconstruction results quantitatively, we define the measurements as follows:

$$bias=\frac{1}{n}\sum_{i=1}^{n}\left(\frac{{\hat{x}}_{i}-x_{i}}{x_{i}}\right)$$ (16)

$$variance=\frac{1}{n-1}\sum_{i=1}^{n}{\left(\frac{{\hat{x}}_{i}-x_{i}}{x_{i}}\right)}^{2}$$ (17)

where *x*_*i*_ is the *i*th pixel of ground truth *x*, ${\hat{x}}_{i}$ is the *i*th pixel of the reconstructed images *x*, since the decomposition of SLCR results in the values of pixel of both stationary and time-varying components are less than the ground truth. For a fair comparison, ${\hat{x}}_{i}$ is defined as the sum of the value of the pixels in the stationary and time-varying components.

Furthermore, we also compute the contrast recovery coefficient (CRC), which is defined as follow:

$$CRC=\frac{Contrast_{measure}}{Contrast_{theory}}=\frac{{\left(S/B\right)}_{measure}-1}{{\left(S/B\right)}_{theory}-1}$$ (18)

where *S* is the mean activity of the region of interest and *B* is the mean activity of the white matter region (background) in the reconstructed image. CRC is used to indicate the contrast of the region of interest in reconstructed images.

### Zubal-thorax Experiment

**Fig 1:**
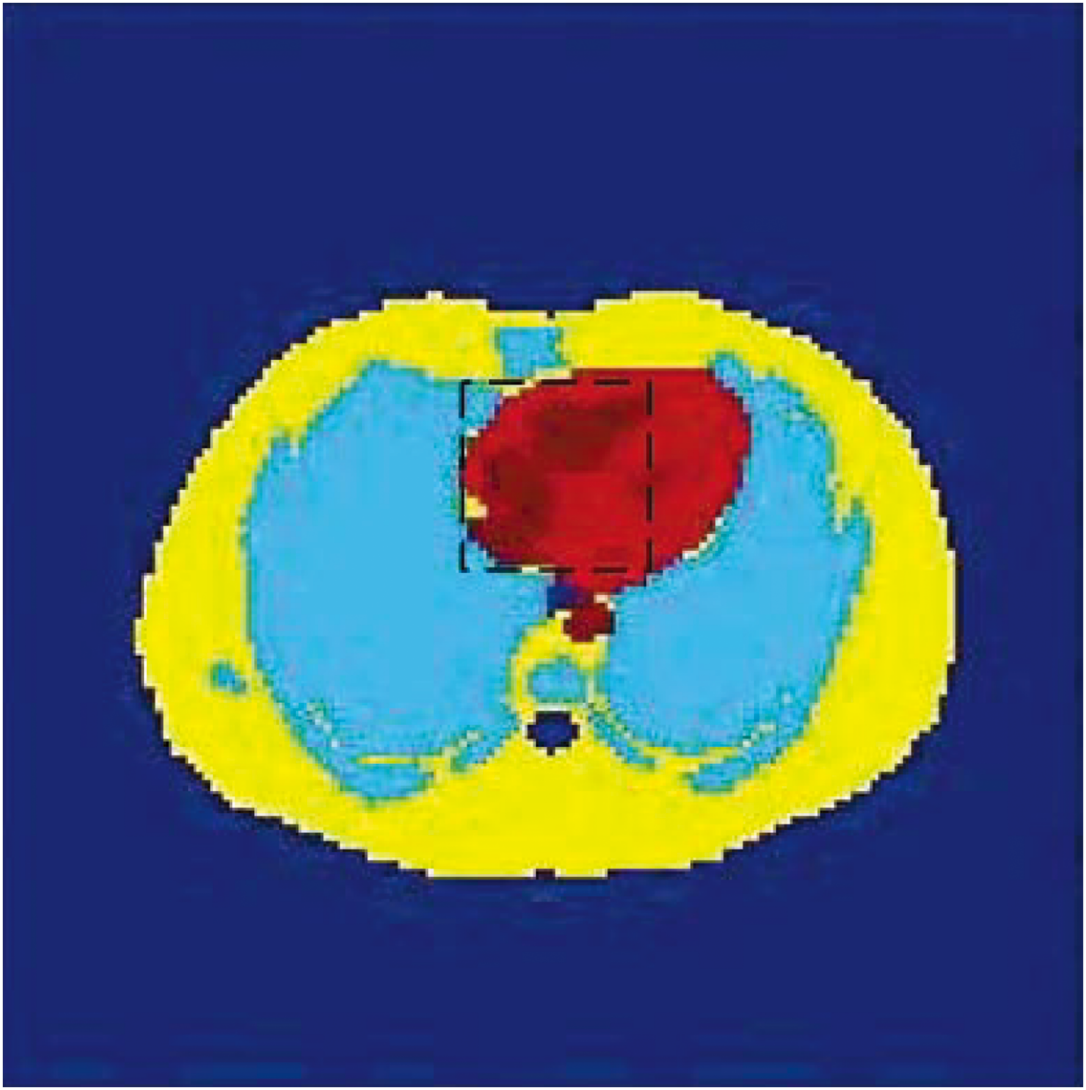
The image of the Zubal-thorax phantom. The target region is marked by black rectangle in phantom.

In the first experiment, the schematic representation of the Zubal-thorax phantom is given in Fig 1. It includes four main regions of interest (ROIs) with different colors. Yellow, red, deep red and soft blue were used to indicate ROI1, ROI2, ROI3 and ROI4 respectively. The deep red region is the target region (marked in black rectangle in phantom and red rectangle in reconstructed images) and this region is the major dynamic part in zubal phantom.

**Fig 2:**
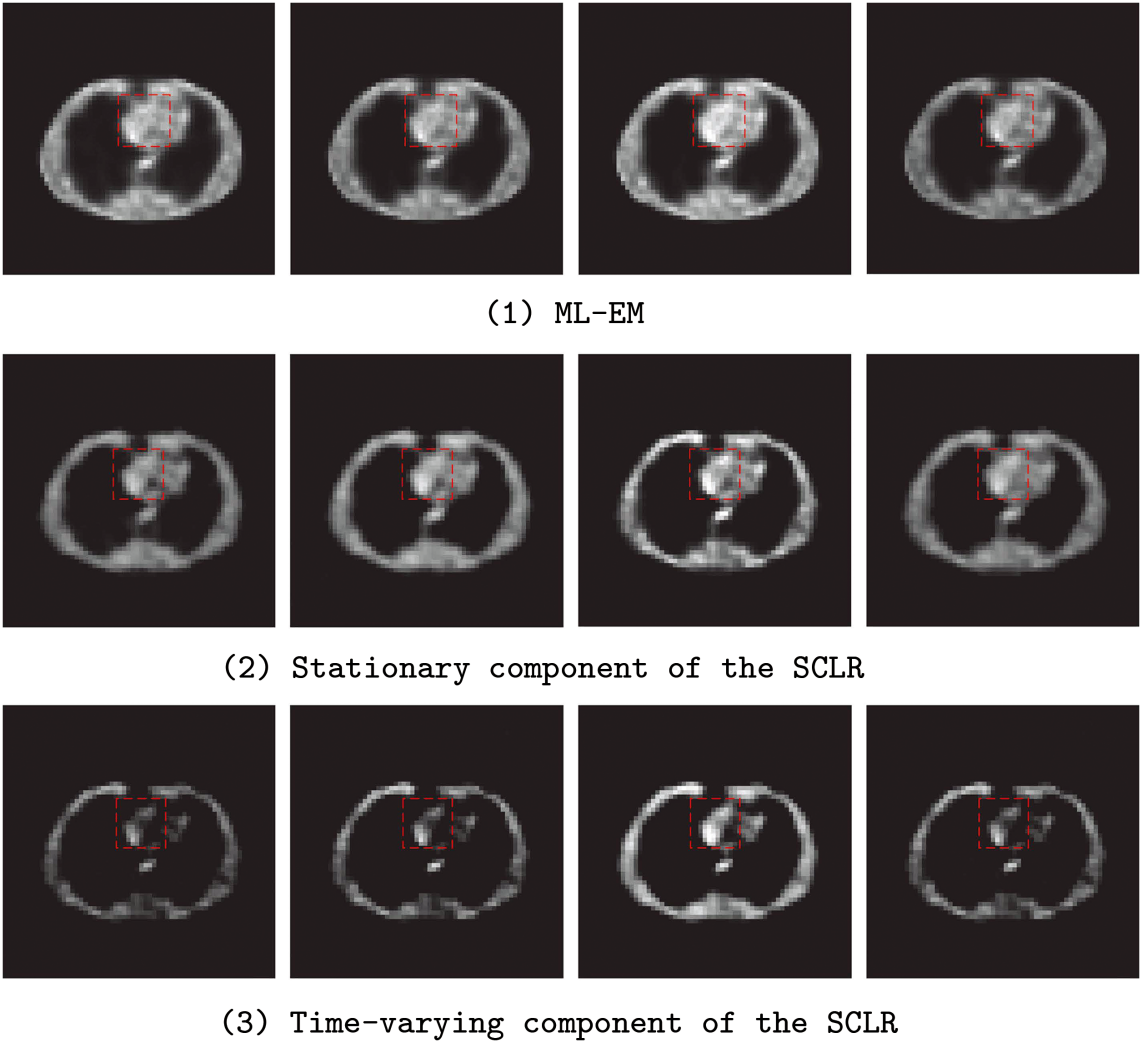
The reconstruction images for the Zubal-thorax phantom. Form the top to bottom, the images in the first line (from left to right is frame #1, 17, 33, 49) are the reconstruction results of the ML-EM method, the second line are the stationary component of SLCR reconstruction images and the third line are the time-varying component of the SLCR reconstruction images.

The simulated PET scanner was Hamamatsu SHR74000 from Hamamatsu Photonics K.K. The radioactivity tracer was C11-acetate, total scanning time was 38 mins, and divided into 53 frames (only results of # 1, 17, 33, 49 frames are shown in figures), all the device settings are the same as in application, including dead time, energy resolution, time resolution and energy window et.al. The images reconstructed by ML-EM (Fig 2(1)) and SLCR method (Fig 2(2) is the stationary component of SLCR and Fig 2(3) is the time-varying component) are given in Fig 2.

In Fig 2(1), there are aliasing artifacts in the reconstruction images of the ML-EM method. It is difficult to distinguish the target region and detail boundary information from adjacent ROIs, even worse, the differences between different frames (from #1 to 20) are not distinctive. Compared with the results of the ML-EM, the results of the SLCR present an obvious reduction of the aliasing artifacts and improve the contrast in the target region (Fig 2(2) and Fig 2(3)). In the meantime, the results of time-varying component indicate the variations between adjacent frames, and it extracts successfully the target region information from the dynamic data set (Fig 2(3)). Moreover, in time-varying images, the radio-activity variation between different frames could be observed easily. It is obvious that the SLCR could provide more helpful information than the ML-EM method.

### Brain Experiment

**Fig 3:**
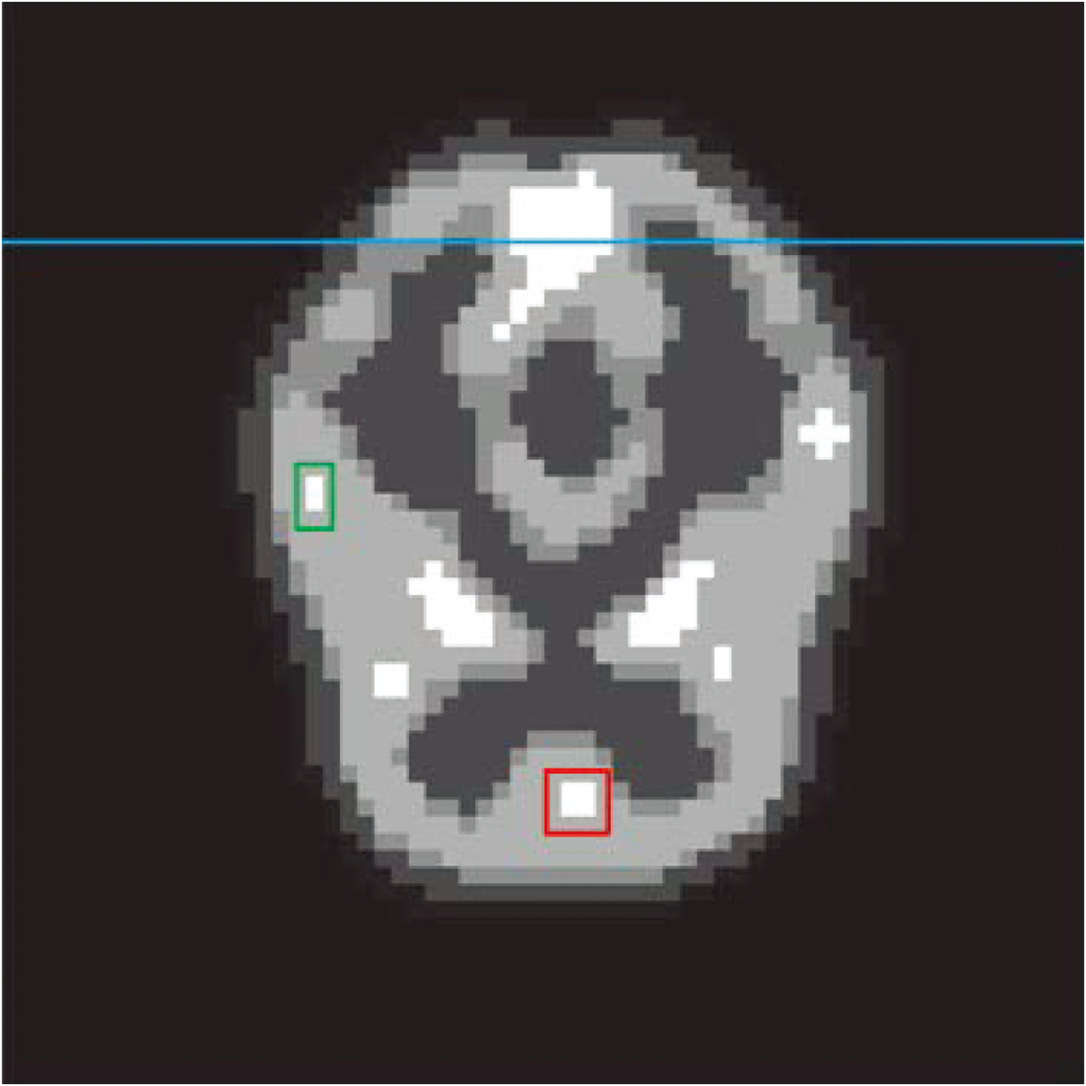
The ground truth of the brain phantom. The target regions are marked by red and green rectangles. And the blue line marks the lateral displacement profile.

**Fig 4:**
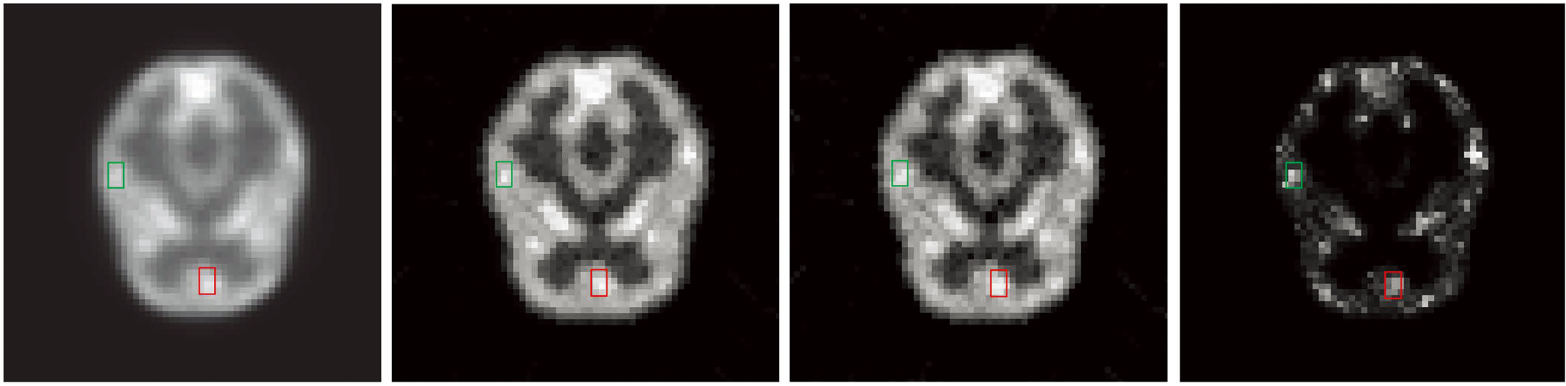
The reconstruction images for brain phantom for count level 1. From left to right, the reconstruction images are the results of the ML-EM (First), the sum of two components of SLCR (ST + SP, second), the stationary (ST, third) and time-varying (SP, forth) components of the SLCR for the brain phantom at the #10 frame in count Level 1.

**Fig 5:**
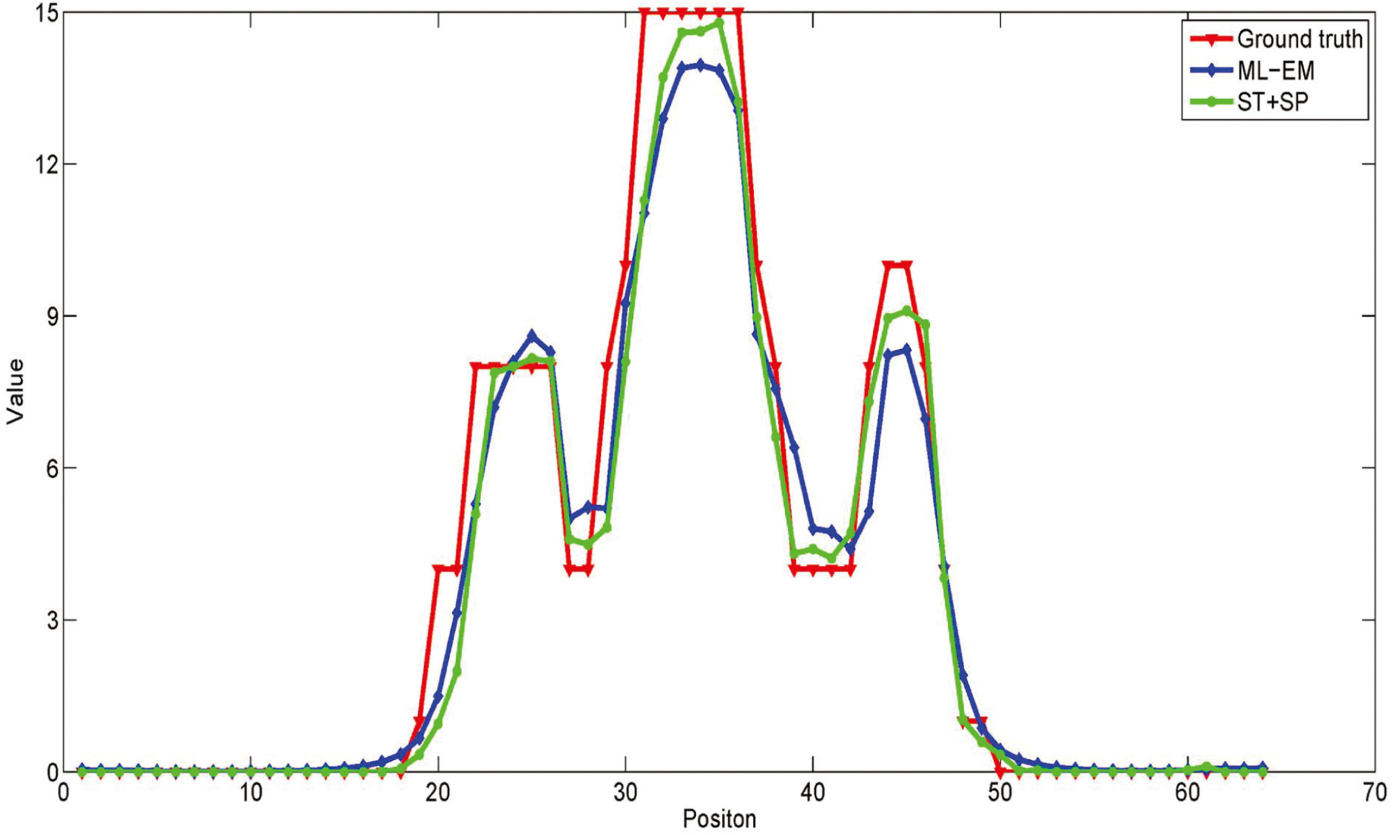
The profile of the reconstruction result. The reconstruction profiles through the marked lines (blue line) in Fig 3.

The data set based on Hoffman brain phantom (Fig 3) was simulated by Monte Carlo simulation in the second experiment. This phantom contains complicated physical structural information and eight highlight areas, the areas marked by red and green rectangle are target areas which present two tumors in human brain, and the reconstruction pixel value of these regions are used for quantitative analysis. The blue line marks the lateral displacement profile. The spatial resolution of the simulated scanner was 3.5 mm full width at half maximum (FWHM) in sagittal or coronal plane and 3.2 mm FWHM in axial plane. The radioactivity tracer was fluorodeoxyglucose (FDG), and the concentration was 333–444 MBq (9–12 mCi/cc). The total scanning time was 30 mins. The data set was divided into 20 frames. The total count of the recorded event was 1.92 × 10^7^ (count level 1) in this data set, the proportion of the scatter events was 0.12%, and the proportion of the random events was 0.063%. The images reconstructed by ML-EM and SLCR (the sum of two components of SLCR (ST + SP), the stationary (ST) and time-varying (SP) components) methods for the #10 frames are shown in Fig 4. And Fig 5 shows the profiles of reconstruction results by ML-EM and SLCR compared with the ground truth. For a fair comparison, the sum of stationary and time-varying components was used in this profiles. It is clear that the SLCR gives the closer fit to ground truth. In Fig 4, the stationary component extracts the background of brain phantom data set, and the time-varying component extracts the dynamic information. It lists the bias and variances and CRC of images reconstructed by ML-EM and SLCR in Table 1. The calculated biases and variances shows that the SLCR provides a more accurate reconstruction than ML-EM. The values of CRC shows that the decomposition of SLCR results in improving the contrast in time-varying component.

**Table 1:** Comparative studies of estimated activity distribution in the red and green rectangle region in brain on synthetic data.

	Red rectangle region			Green rectangle region		
Method	Bias	Variance	CRC	Bias	Variance	CRC
ML-EM	0.135	0.067	0.843	0.084	0.054	0.850
SLCR(ST + SP)	0.104	0.054	0.854	0.068	0.049	0.858
Stationary (ST)			0.842			0.849
Time-varying (SP)			0.867			0.868

**Fig 6:**
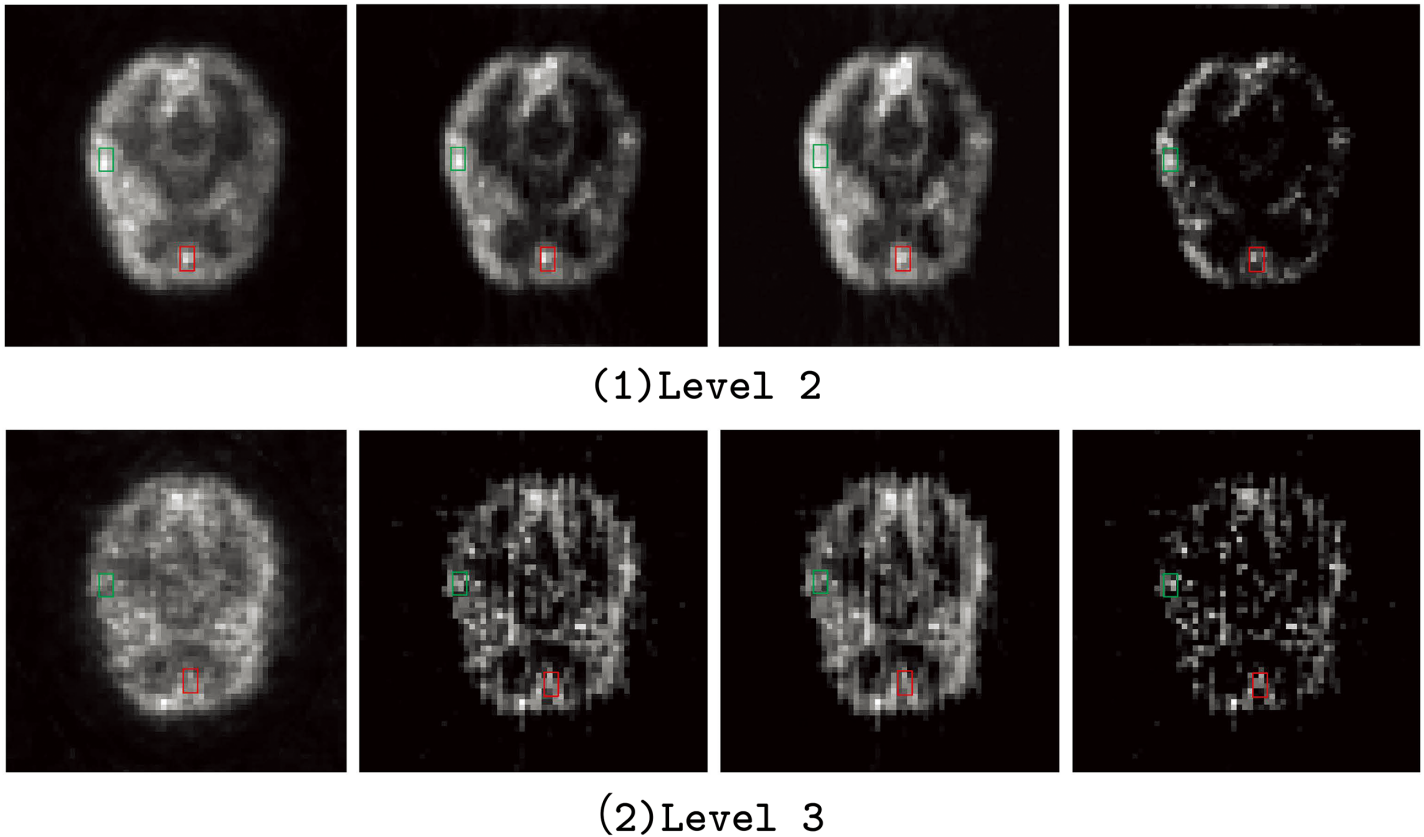
The reconstruction images for brain phantom for count level 2 and 3. From left to right, the reconstruction images are the results of the ML-EM (first), the sum of two components of SLCR (ST + SP, second), the stationary (ST, third) and time-varying (SP, forth) components of the SLCR for the brain phantom at the #10 frame in count Level 2 to 3.

To evaluate the robustness of the SLCR in low count data, two extra count levels were simulated and the corresponding proportions of the random and scatter events were recorded. Level 2: the total count was 1.32 × 10^6^, the proportion of the scatter events was 19.7%, and the proportion of the random events was 1.63%); Level 3: the total count was 6 × 10^5^, the proportion of the scatter events was 39.75%, and the proportion of the random events was 3.363%. Data sets in both level 2 and level 3 are considered as the low-count data. The images reconstructed by ML-EM and SLCR methods for these two levels are shown in Fig 6. Though all images reconstructed by ML-EM and SLCR go worse when the data count decrease, the SLCR method provide more accurate and less aliasing artifacts reconstructions than ML-EM in both count levels, especially in the target regions. In addition, it is easy to locate the position of the target regions in the time-varying component of SLCR.

The bias, variances and CRC of the reconstructions in these two levels are listed in Table 2.

**Table 2:** Comparative studies of estimated activity distribution in the red and green rectangle region in brain for count level 2 and 3.

Count level 2						
	Red rectangle region			Green rectangle region		
Method	Bias	Variance	CRC	Bias	Variance	CRC
ML-EM	0.323	0.189	0.701	0.384	0.168	0.712
SLCR(ST + SP)	0.291	0.162	0.723	0.308	0.155	0.730
Stationary (ST)			0.703			0.716
Time-varying (SP)			0.752			0.767
Count level 3						
	Red rectangle region			Green rectangle region		
Method	Bias	Variance	CRC	Bias	Variance	CRC
ML-EM	0.648	0.478	0.531	0.675	0.522	0.501
SLCR(ST + SP)	0.607	0.401	0.542	0.651	0.499	0.553
Stationary (ST)			0.530			0.481
Time-varying (SP)			0.557			0.619

The quantitative analysis shows that the bias and variance for ML-EM go up faster than SLCR when the data count decrease. All of these demonstrate that the SCLR is better and more robust reconstruction method.

### Cardiac Experiment

**Fig 7:**
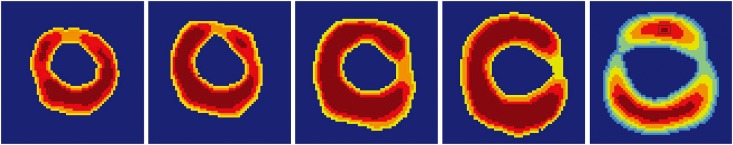
The truth images sequence of the cardiac phantom. The target region is marked by red rectangle in 5th picture.

**Fig 8:**
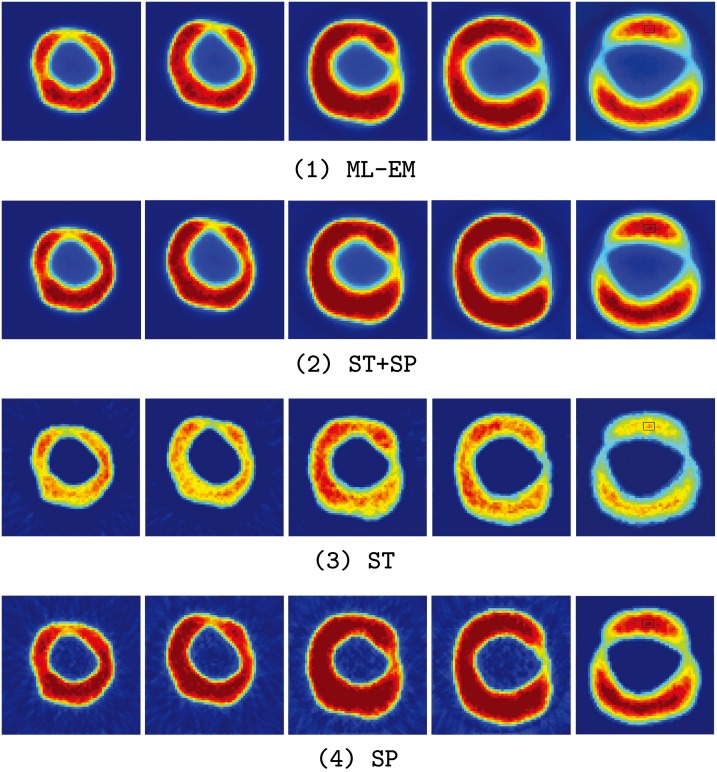
The reconstruction images for the cardiac phantoms in the count Level 1. From the top to bottom, the first line is the images reconstructed by ML-EM, the second line is the images of the sum of stationary and time-varying components of SLCR (ST + SP), the third line is the images of the stationary component (ST) of SLCR, and the last line is the images of the time-varying component (SP) of SLCR.

**Fig 9:**
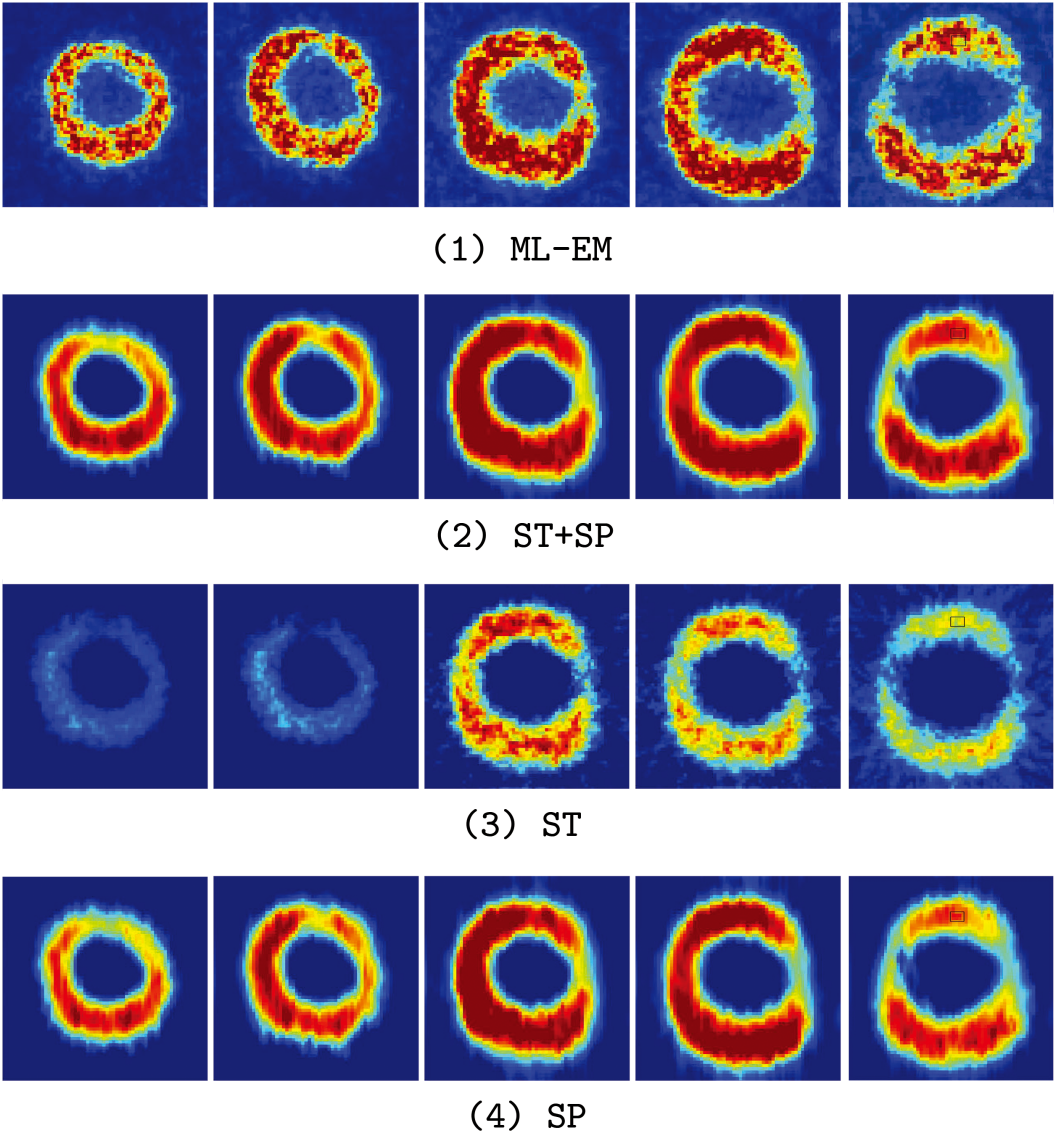
The reconstruction images for the cardiac phantoms in the count Level 2. From the top to bottom, the first line is the images reconstructed by ML-EM, the second line is the images of the sum of stationary and time-varying components of SLCR (ST + SP), the third line is the images of the stationary component of SLCR, and the last line is the images of the time-varying component of SLCR.

In the third experiment, a series of the cardiac phantoms with respected to the short axis of cardiac in stress (only five frames were shown in the results due to the limitation of the number of pages) were simulated. And the region selected for quantitative analysis is marked by the black rectangle in the 5*th* picture. Such a highlight area always indicates a potential lesions or abnormal tissue in clinical situation. These cardiac phantoms were based on a 61-year-old patient with arterial hypertensionand type 2 diabetes mellitus. A distinctive shape deformations (volume variation and myocardial wall motion) were included in these data sets. The main purpose of this experiment was to evaluate the effectiveness of the motion extraction of the SLCR method. The radioactivity tracer was 13*N* − *Ammonia*. In addition, to evaluate the robustness of the SLCR method, two count levels were simulated. The proportions of the scatter and random events in these two levels were recorded, level 1: the total count is 5.4 × 10^6^, and it contains 0.1% scatter events and 0.06% random events. level 2: the total count is 1.2 × 10^5^ (low count data), and it contains 19.54% scatter events and 1.2% random events. Similar to the former two experiments, the ML-EM method is implemented as the comparison. The truth images sequences of the cardiac phantom are given in Fig 7. The images reconstructed by the ML-EM and SLCR in different count levels have been shown in Fig 8 (Level 1) and Fig 9 (Level 2), respectively. From these figures, two conclusions could be concluded:

1. The SLCR method is able to extract the dynamic and structural information effectively when the organ has a large deformation. Since the geometric shape of different frames are quite different in the cardiac sequence, it only contains the outline of the short axis of cardiac in the stationary component and loses lots of detailed information in the marginal area of the different regions. In contrast, the detailed information in the marginal area of the different regions are enhanced in the time-varying component. Since the decomposition of the SLCR enhances the structural and marginal information in stationary and time-varying components respectively, the sum of these two components shows a more accurate reconstructions.
2. The SLCR also has a good reconstruction result even for the low count data. When data count is not sufficient, the results of the ML-EM method are heavily corrupted by the random/scatter events. There are aliasing artifacts and noise points in the reconstruction images of the ML-EM method. The SLCR method solved this problem. Though the image quality of the SLCR method also deteriorates in the low count data, the results of both stationary and time-varying components show a more clear outline and detailed information than ML-EM. In addition, the sum of these two components of SLCR provides the better reconstruction. Therefore, the SCLR could provide a better reconstruction results than ML-EM in any count levels.

The bias, variance and CRC in the target region are calculated and shown in Table 3. The quantitative analysis results shows that the bias and variance for ML-EM go up faster than SLCR when the data count decreases. And the SLCR could provide more accurate and robust reconstruction images than ML-EM in different count levels data. All of these demonstrate that the SCLR is better and more robust reconstruction method. In addition, the values of CRC also shows that the decomposition of SLCR results in the improvement of the contrast in time-varying component. It is easy to locate the position of the target regions in the SLCR especially in the time-varying component.

**Table 3:** Comparative studies of estimated activity distribution in heart on synthetic data for different count levels.

Noise Level	Method	Bias	Variance	CRC
level 1	ML-EM	0.268	0.128	0.857
	SLCR (ST + SP)	0.224	0.103	0.868
	Stationary (ST)			0.836
	Time-varying (SP)			0.887
level 2	ML-EM	0.473	0.354	0.625
	SLCR	0.449	0.326	0.643
	Stationary (ST)			0.613
	Time-varying (SP)			0.667

## Discussion

Summarizing all results of the three experiments, the SCLR is capable of extracting the background and dynamic components during reconstruction, and producing a high contrast and accurate reconstruction images even in a low count data. Compared with the conventional ML-EM method, both of the accuracy and robustness of images are improved. However, the interference or information sharing between background and time-varying components caused by strong coherence between the adjoint frames exists in all experiments, especially when the difference between adjoint frames is not remarkable. One possible reason for this phenomenon is that the sparsity constraint in the framelet domain is not enough to force the time-varying component only contains dynamic information. In the future work, we will focus on how to solve this problem.

## Conclusion

A novel method called SLCR is proposed, analyzed and tested for dynamic PET imaging in this work. The advantages of this method are that 1) it is capable of separating the background and dynamic components during reconstruction, 2) it is able to afford a high contrast and accurate reconstruction images even in a low count data. Three experiments have been used to demonstrate the effectiveness and robustness of the SLCR method. However, for a real clinical application of dynamic PET imaging such as monitoring of radiotherapy, more work needs to be done. For example, the rate of convergence in SCLR is still too slow, the maximum number of iteration is too large for a clinical application. In addition, the interference between stationary background and time-varying components also needs to be solved. Therefore, we will cope with these problems in the future work.
